# *N*-Ethyl-3,5-dinitro­benzamide

**DOI:** 10.1107/S160053681100804X

**Published:** 2011-03-09

**Authors:** Jia-Ying Xu, Wei-Hua Cheng

**Affiliations:** a College of Chemical and Biological Engineering, Yancheng Institute of Technology, Yinbing Road No. 9 Yancheng, Yancheng 224051, People’s Republic of China; b Department of Chemical Engineering, Yancheng College of Textile Technology, Yancheng 224051, People’s Republic of China

## Abstract

In the title mol­ecule, C_9_H_9_N_3_O_5_, the dihedral angle between the mean planes of the amide group and the benzene ring is 31.24 (14)°. In the crystal, N—H⋯O hydrogen bonds link the mol­ecules to form one-dimensional chains propagating in [100].

## Related literature

For the synthesis of the title compound, see: Lee *et al.* (2009 ▶). For standard bond-length data, see: Allen *et al.* (1987 ▶).
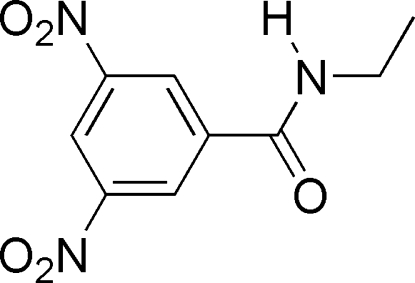


## Experimental

### 

#### Crystal data

C_9_H_9_N_3_O_5_
*M* *_r_* = 239.19
Triclinic, 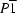

*a* = 4.854 (1) Å
*b* = 10.488 (2) Å
*c* = 10.851 (2) Å
α = 101.49 (3)°
β = 97.84 (3)°
γ = 95.25 (3)°
*V* = 532.26 (19) Å^3^
*Z* = 2
Mo *K*α radiation
μ = 0.12 mm^−1^
*T* = 293 K
0.20 × 0.10 × 0.10 mm

#### Data collection

Enraf–Nonius CAD-4 diffractometer
Absorption correction: ψ scan (North *et al.*, 1968 ▶) *T*_min_ = 0.976, *T*_max_ = 0.988
2203 measured reflections
1955 independent reflections
1321 reflections with *I* > 2σ(*I*)
*R*_int_ = 0.021
3 standard reflections every 200 reflections  intensity decay: 1%

#### Refinement

*R*[*F* ^2^ > 2σ(*F* ^2^)] = 0.051
*wR*(*F* ^2^) = 0.161
*S* = 1.01
1955 reflections
154 parameters
H-atom parameters constrained
Δρ_max_ = 0.15 e Å^−3^
Δρ_min_ = −0.23 e Å^−3^

### 

Data collection: *CAD-4 Software* (Enraf–Nonius, 1985 ▶); cell refinement: *CAD-4 Software*; data reduction: *XCAD4* (Harms & Wocadlo,1995 ▶); program(s) used to solve structure: *SHELXS97* (Sheldrick, 2008 ▶); program(s) used to refine structure: *SHELXL97* (Sheldrick, 2008 ▶); molecular graphics: *SHELXTL* (Sheldrick, 2008 ▶); software used to prepare material for publication: *SHELXTL*.

## Supplementary Material

Crystal structure: contains datablocks I, global. DOI: 10.1107/S160053681100804X/su2259sup1.cif

Structure factors: contains datablocks I. DOI: 10.1107/S160053681100804X/su2259Isup2.hkl

Additional supplementary materials:  crystallographic information; 3D view; checkCIF report

## Figures and Tables

**Table 1:** Hydrogen-bond geometry (Å, °)

*D*—H⋯*A*	*D*—H	H⋯*A*	*D*⋯*A*	*D*—H⋯*A*
N3—H3*A*⋯O5^i^	0.86	2.13	2.886 (3)	146

Symmetry code: (i) 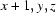.

**Fig. 1.**
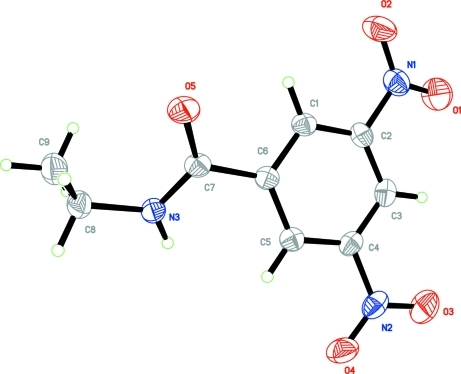
The molecular structure of the title molecule, with displacement ellipsoids drawn at the 50% probability level.

**Fig. 2.**
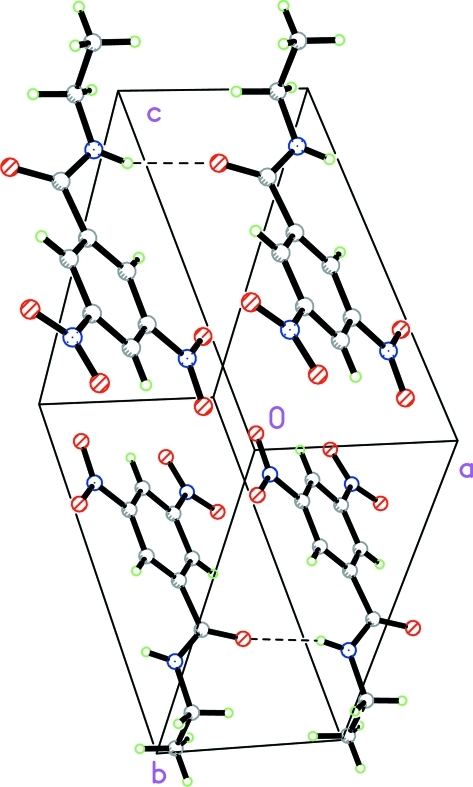
A view of the crystal packing of the title compound. The N—H···O hydrogen bonds are shown as dashed lines.

## supplementary crystallographic information

### Comment

The title compound is an important organic intermediate, and such amide derivatives exhibit biological activities, such as antibacterial and antifungal effects (Lee *et al.*, 2009). Herein we report on the crystal structure of the title substitued benzamide compound.

The molecular structure of the title compound is illustrated in Fig. 1. The bond lengths and angles are within normal ranges (Allen *et al.*, 1987). The dihedral angle between the amide group [atoms O5,N3,C7,C8, planar to within 0.012 Å] and the benzene ring (C1-C6) is 31.24 (14) °.

In the crystal of the title compound molecules are connected *via* N—H···O intermolecular hydrogen bonds (Table 1), to form a one-dimensional polymer propagating in [100]. These chains stack along the *c* axis direction.

### Experimental

The title compound was prepared following a literature proceedure (Lee *et al.*, 2009). Crystals, suitable for X-ray diffraction analysis, were obtained by slow evaporation, over a period of 5 days, of a solution of the title compound in ethanol [0.2 g, 0.84 mmol in 25 ml ethanol].

### Refinement

All the H-atoms were positioned geometrically and constrained to ride on their parent atom: N-H = 0.86 Å, C—H = 0.93, 0.96 and 0.97 Å for CH(aromatic), CH_2_ and CH_3_ H-atoms, respectively, with *U*_iso_(H) = k × *U*_eq_(C or N), where *k* = 1.5 for CH_3_ H-atoms, and k = 1.2 for all other H-atoms.

### Crystal data

**Table:**

C_9_H_9_N_3_O_5_	*Z* = 2
*M**_r_* = 239.19	*F*(000) = 248
Triclinic, *P*1	*D*_x_ = 1.492 Mg m^−^^3^
Hall symbol: -P 1	Mo *K*α radiation, λ = 0.71073 Å
*a* = 4.854 (1) Å	Cell parameters from 25 reflections
*b* = 10.488 (2) Å	θ = 10–13°
*c* = 10.851 (2) Å	µ = 0.12 mm^−^^1^
α = 101.49 (3)°	*T* = 293 K
β = 97.84 (3)°	Block, colourless
γ = 95.25 (3)°	0.20 × 0.10 × 0.10 mm
*V* = 532.26 (19) Å^3^	

### Data collection

**Table:**

Enraf–Nonius CAD-4 diffractometer	1321 reflections with *I* > 2σ(*I*)
Radiation source: fine-focus sealed tube	*R*_int_ = 0.021
graphite	θ_max_ = 25.4°, θ_min_ = 1.9°
ω/2θ scans	*h* = 0→5
Absorption correction: ψ scan (North *et al.*, 1968)	*k* = −12→12
*T*_min_ = 0.976, *T*_max_ = 0.988	*l* = −13→12
2203 measured reflections	3 standard reflections every 200 reflections
1955 independent reflections	intensity decay: 1%

### Refinement

**Table:**

Refinement on *F*^2^	Primary atom site location: structure-invariant direct methods
Least-squares matrix: full	Secondary atom site location: difference Fourier map
*R*[*F*^2^ > 2σ(*F*^2^)] = 0.051	Hydrogen site location: inferred from neighbouring sites
*wR*(*F*^2^) = 0.161	H-atom parameters constrained
*S* = 1.01	*w* = 1/[σ^2^(*F*_o_^2^) + (0.090*P*)^2^] where *P* = (*F*_o_^2^ + 2*F*_c_^2^)/3
1955 reflections	(Δ/σ)_max_ < 0.001
154 parameters	Δρ_max_ = 0.15 e Å^−^^3^
0 restraints	Δρ_min_ = −0.22 e Å^−^^3^

### Special details

**Table:**

Geometry. All e.s.d.'s (except the e.s.d. in the dihedral angle between two l.s. planes) are estimated using the full covariance matrix. The cell e.s.d.'s are taken into account individually in the estimation of e.s.d.'s in distances, angles and torsion angles; correlations between e.s.d.'s in cell parameters are only used when they are defined by crystal symmetry. An approximate (isotropic) treatment of cell e.s.d.'s is used for estimating e.s.d.'s involving l.s. planes.
Refinement. Refinement of *F*^2^ against ALL reflections. The weighted *R*-factor *wR* and goodness of fit *S* are based on *F*^2^, conventional *R*-factors *R* are based on *F*, with *F* set to zero for negative *F*^2^. The threshold expression of *F*^2^ > σ(*F*^2^) is used only for calculating *R*-factors(gt) *etc*. and is not relevant to the choice of reflections for refinement. *R*-factors based on *F*^2^ are statistically about twice as large as those based on *F*, and *R*- factors based on ALL data will be even larger.

### Fractional atomic coordinates and isotropic or equivalent isotropic displacement parameters (Å^2^)

**Table:**

	*x*	*y*	*z*	*U*_iso_*/*U*_eq_	
C1	0.1490 (5)	0.7075 (2)	0.2465 (2)	0.0428 (6)	
H1A	−0.0017	0.6473	0.2494	0.051*	
N1	0.0031 (5)	0.6713 (2)	0.0152 (2)	0.0617 (6)	
O1	0.0675 (6)	0.6862 (3)	−0.0856 (2)	0.0985 (9)	
N2	0.8248 (5)	0.9842 (2)	0.2289 (3)	0.0576 (6)	
C2	0.1956 (5)	0.7377 (2)	0.1327 (2)	0.0454 (6)	
O2	−0.2072 (5)	0.6034 (2)	0.02613 (19)	0.0795 (7)	
N3	0.4868 (4)	0.7427 (2)	0.57021 (18)	0.0453 (5)	
H3A	0.6513	0.7677	0.5563	0.054*	
C3	0.4127 (5)	0.8280 (2)	0.1236 (2)	0.0492 (6)	
H3B	0.4392	0.8480	0.0459	0.059*	
O3	0.8469 (5)	1.0119 (2)	0.1269 (2)	0.0911 (8)	
C4	0.5890 (5)	0.8874 (2)	0.2352 (2)	0.0449 (6)	
O4	0.9911 (4)	1.0292 (2)	0.3254 (2)	0.0743 (6)	
O5	0.0241 (3)	0.70717 (19)	0.49151 (17)	0.0574 (5)	
C5	0.5555 (5)	0.8591 (2)	0.3510 (2)	0.0414 (6)	
H5A	0.6803	0.8997	0.4241	0.050*	
C6	0.3318 (4)	0.7688 (2)	0.3573 (2)	0.0388 (5)	
C7	0.2693 (4)	0.7366 (2)	0.4798 (2)	0.0408 (6)	
C8	0.4582 (5)	0.7087 (2)	0.6918 (2)	0.0497 (6)	
H8A	0.2783	0.7293	0.7144	0.060*	
H8B	0.6035	0.7612	0.7572	0.060*	
C9	0.4794 (7)	0.5668 (3)	0.6881 (3)	0.0712 (9)	
H9A	0.4582	0.5484	0.7697	0.107*	
H9B	0.6592	0.5465	0.6680	0.107*	
H9C	0.3345	0.5145	0.6242	0.107*	

### Atomic displacement parameters (Å^2^)

**Table:**

	*U*^11^	*U*^22^	*U*^33^	*U*^12^	*U*^13^	*U*^23^
C1	0.0323 (12)	0.0467 (13)	0.0504 (14)	0.0078 (10)	0.0086 (10)	0.0101 (11)
N1	0.0608 (16)	0.0709 (16)	0.0496 (14)	0.0068 (13)	0.0017 (11)	0.0094 (11)
O1	0.113 (2)	0.131 (2)	0.0458 (13)	−0.0138 (16)	0.0034 (12)	0.0245 (13)
N2	0.0512 (14)	0.0510 (13)	0.0797 (17)	0.0095 (11)	0.0225 (13)	0.0255 (12)
C2	0.0424 (14)	0.0518 (15)	0.0425 (13)	0.0125 (11)	0.0041 (10)	0.0101 (11)
O2	0.0575 (13)	0.1052 (18)	0.0627 (13)	−0.0106 (12)	−0.0020 (10)	0.0039 (12)
N3	0.0292 (10)	0.0650 (13)	0.0446 (11)	0.0026 (9)	0.0098 (8)	0.0172 (9)
C3	0.0528 (15)	0.0544 (15)	0.0489 (14)	0.0174 (12)	0.0161 (12)	0.0207 (12)
O3	0.0941 (18)	0.1021 (18)	0.0914 (17)	−0.0079 (14)	0.0313 (14)	0.0522 (14)
C4	0.0396 (13)	0.0423 (13)	0.0586 (15)	0.0087 (10)	0.0153 (11)	0.0176 (11)
O4	0.0597 (13)	0.0651 (13)	0.0940 (16)	−0.0137 (10)	0.0088 (12)	0.0193 (11)
O5	0.0287 (9)	0.0861 (14)	0.0596 (11)	0.0045 (8)	0.0149 (8)	0.0164 (9)
C5	0.0330 (12)	0.0439 (13)	0.0489 (13)	0.0095 (10)	0.0090 (10)	0.0098 (11)
C6	0.0287 (11)	0.0446 (13)	0.0458 (13)	0.0100 (10)	0.0090 (9)	0.0114 (10)
C7	0.0270 (12)	0.0501 (14)	0.0468 (13)	0.0074 (10)	0.0115 (10)	0.0089 (10)
C8	0.0454 (14)	0.0633 (17)	0.0415 (14)	0.0050 (12)	0.0099 (11)	0.0125 (12)
C9	0.089 (2)	0.0671 (19)	0.0588 (18)	0.0082 (17)	0.0087 (16)	0.0185 (15)

### Geometric parameters (Å, °)

**Table:**

C1—C2	1.376 (3)	C3—C4	1.380 (4)
C1—C6	1.394 (3)	C3—H3B	0.9300
C1—H1A	0.9300	C4—C5	1.375 (3)
N1—O1	1.212 (3)	O5—C7	1.232 (3)
N1—O2	1.224 (3)	C5—C6	1.392 (3)
N1—C2	1.479 (3)	C5—H5A	0.9300
N2—O4	1.214 (3)	C6—C7	1.498 (3)
N2—O3	1.214 (3)	C8—C9	1.494 (4)
N2—C4	1.477 (3)	C8—H8A	0.9700
C2—C3	1.379 (4)	C8—H8B	0.9700
N3—C7	1.327 (3)	C9—H9A	0.9600
N3—C8	1.454 (3)	C9—H9B	0.9600
N3—H3A	0.8600	C9—H9C	0.9600
			
C2—C1—C6	118.8 (2)	C4—C5—C6	119.0 (2)
C2—C1—H1A	120.6	C4—C5—H5A	120.5
C6—C1—H1A	120.6	C6—C5—H5A	120.5
O1—N1—O2	124.6 (2)	C5—C6—C1	119.7 (2)
O1—N1—C2	117.6 (2)	C5—C6—C7	123.0 (2)
O2—N1—C2	117.8 (2)	C1—C6—C7	117.3 (2)
O4—N2—O3	123.4 (2)	O5—C7—N3	124.0 (2)
O4—N2—C4	118.2 (2)	O5—C7—C6	119.2 (2)
O3—N2—C4	118.4 (3)	N3—C7—C6	116.78 (19)
C1—C2—C3	122.9 (2)	N3—C8—C9	112.1 (2)
C1—C2—N1	118.7 (2)	N3—C8—H8A	109.2
C3—C2—N1	118.5 (2)	C9—C8—H8A	109.2
C7—N3—C8	122.68 (19)	N3—C8—H8B	109.2
C7—N3—H3A	118.7	C9—C8—H8B	109.2
C8—N3—H3A	118.7	H8A—C8—H8B	107.9
C2—C3—C4	116.8 (2)	C8—C9—H9A	109.5
C2—C3—H3B	121.6	C8—C9—H9B	109.5
C4—C3—H3B	121.6	H9A—C9—H9B	109.5
C5—C4—C3	122.7 (2)	C8—C9—H9C	109.5
C5—C4—N2	118.9 (2)	H9A—C9—H9C	109.5
C3—C4—N2	118.3 (2)	H9B—C9—H9C	109.5
			
C6—C1—C2—C3	−1.3 (4)	C3—C4—C5—C6	−1.3 (4)
C6—C1—C2—N1	179.3 (2)	N2—C4—C5—C6	179.42 (19)
O1—N1—C2—C1	−170.8 (3)	C4—C5—C6—C1	0.9 (3)
O2—N1—C2—C1	8.0 (4)	C4—C5—C6—C7	−176.8 (2)
O1—N1—C2—C3	9.8 (4)	C2—C1—C6—C5	0.3 (3)
O2—N1—C2—C3	−171.4 (2)	C2—C1—C6—C7	178.2 (2)
C1—C2—C3—C4	0.9 (4)	C8—N3—C7—O5	3.0 (4)
N1—C2—C3—C4	−179.6 (2)	C8—N3—C7—C6	−177.4 (2)
C2—C3—C4—C5	0.4 (4)	C5—C6—C7—O5	147.8 (2)
C2—C3—C4—N2	179.7 (2)	C1—C6—C7—O5	−30.0 (3)
O4—N2—C4—C5	4.5 (3)	C5—C6—C7—N3	−31.9 (3)
O3—N2—C4—C5	−177.4 (2)	C1—C6—C7—N3	150.3 (2)
O4—N2—C4—C3	−174.8 (2)	C7—N3—C8—C9	90.0 (3)
O3—N2—C4—C3	3.2 (3)		

### Hydrogen-bond geometry (Å, °)

**Table:**

*D*—H···*A*	*D*—H	H···*A*	*D*···*A*	*D*—H···*A*
N3—H3A···O5^i^	0.86	2.13	2.886 (3)	146

Symmetry codes: (i) *x*+1, *y*, *z*.
